# Favorable outcomes of NPM1^mut^ AML patients are due to transcriptional inactivation of FOXM1, presenting a new target to overcome chemoresistance

**DOI:** 10.1038/s41408-023-00898-4

**Published:** 2023-08-22

**Authors:** I. Khan, A. Kaempf, S. Raghuwanshi, M. Chesnokov, X. Zhang, Z. Wang, A. Domling, J. W. Tyner, C. Camacho, A. L. Gartel

**Affiliations:** 1 University of Illinois at Chicago, Department of Medicine, Chicago, IL USA; 2 Robert H Lurie Comprehensive Cancer Center, Northwestern University, Chicago, IL USA; 3 OHSU Knight Cancer Institute, School of Medicine, Portland, OR USA; 4 Centro Nacional de Investigaciones Oncológicas (CNIO), Madrid, Spain; 5 The Czech Advanced Technology and Research Institute (CATRIN) of Palacký University, Olomouc, Czech Republic; 6 University of Groningen, Groningen, Netherlands; 7 Computational and Systems Biology, University of Pittsburgh, Pittsburgh, PA USA

**Keywords:** Acute myeloid leukaemia, Acute myeloid leukaemia

**Dear Editor**,

Acute Myeloid Leukemia (AML) is a highly heterogeneous disease with 3-year patient survival ranging from 30 to 80%, depending on molecular characteristics. Mutations in nucleophosmin (NPM1^mut^), identified in a fourth of AML cases, are highly deterministic of treatment response [1]. The paradigm that clinically relevant NPM1 mutations aberrantly relocalize the NPM protein to the cytoplasm, producing favorable clinical outcomes, was first reported in 2005 . The NPM1 mutation confers superior responses to induction chemotherapy, as confirmed by multiple cooperative groups, and favorable responses to the BCL2 inhibitor, venetoclax [2]. However, the mechanistic basis for this treatment sensitization remains obscure. Our lab previously identified NPM1 as a binding partner of forkhead box M1 (FOXM1) and a critical determinant of FOXM1 cellular localization by performing reciprocal pull-down experiments, mass spectrometry, and confocal imaging on cell lines [3] and patient samples [4]. FOXM1 is a Forkhead family transcription factor that has been implicated in all the hallmarks of cancer. PRECOG meta-analysis identified the FOXM1 regulatory network as a major predictor of adverse outcomes across 30 tumor histologies [5]. We have previously demonstrated that knockdown of FOXM1 in AML cells and animal models of leukemia modulates sensitivity to chemotherapy and bcl2 inhibitors [6, 7].

This has fueled the discovery by many labs, including ours, of direct and indirect pharmacological FOXM1 inhibitors, including the proteasome inhibitors, such as thiostrepton, bortezomib, and ixazomib [8], honokiol [9], FDI-6 [10], RCM-1 [11], peptides, and more recently PROTACs. However, clinical advancement has been thwarted by the unknown inhibitory pathways of these compounds, pharmacokinetic issues, and off-target activity. In the current study, we firstly sought to assess the correlation between a FOXM1 transcriptional signature and NPM1 mutational status and its independent prognostic significance using the OHSU Beat AML database [12] and, secondly, we investigated a novel small molecule FOXM1 inhibitor that recapitulates the nuclear export of FOXM1 to potentiate the effects of standard AML clinical therapies.

**Fig. 1:**
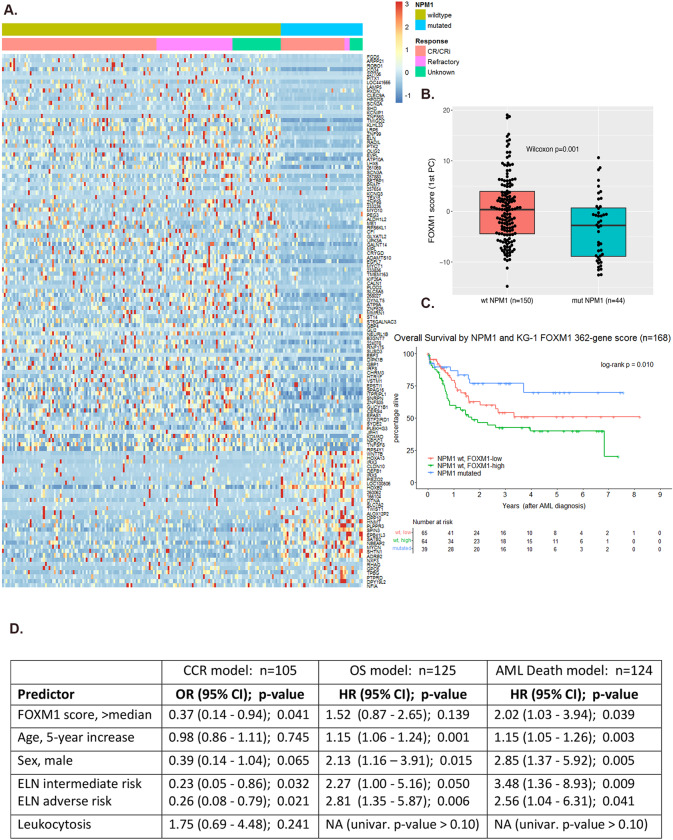
FOXM1 is transcriptionally downregulated in NPM1 mutant AML and is an independent predictor of chemotherapy response and disease-specific survival. **A** Heatmap of gene(row)-standardized RNAseq gene expression values of 125 FOXM1 signature genes for de novo, FLT3-wildtype AML patients (*n* = 194 patients; NPM1^wt^ = 150; NPM1^mut^ = 44). Included genes had |log2 FC| >1 on differential expression analysis of NPM1-mutated vs. NPM1-wildtype patient samples. From top to bottom, genes are ordered by decreasing |FC|, with the 93 genes downregulated in NPM1-mutant samples preceding the 32 genes upregulated in NPM1-mutant samples. **B** Dot and box plot of FOXM1 signature scores (i.e., 1^st^ principal components of RNAseq normalized read counts for 362 genes identified by our prior KG-1 shRNA experiment) by NPM1 status in de novo, FLT3-wildtype AML patients from the Beat AML cohort. The difference in FOXM1 scores by NPM1 group was assessed by the Wilcoxon rank sum test. (*n* = 194 patients). **C** Overall survival Kaplan-Meier curves for 168 de novo, FLT3-wildtype AML patients treated with standard induction chemotherapy when categorized by NPM1 status and, for NPM1-wildtype patients, FOXM1 transcriptional score (derived from the 362 genes identified from our prior KG-1 shRNA experiment) dichotomized at the sample median. **D** Multivariable model results for CCR (logistic regression), OS (Cox regression), and disease-related death (Fine-Gray regression). Included Beat AML patients are de novo, FLT3-wildtype, NPM1-wildtype AML treated with standard induction chemotherapy. In the presence of age, sex, leukocytosis and ELN risk score, increased FOXM1 activity remained a potent negative predictor of composite complete remission following chemotherapy (HR 0.37) and inferior survival with a 2-fold increased risk of leukemia-related death (HR = 2.02). Abbreviations: *n* = multivariable model sample size, CCR = Composite Complete Remission, OS = Overall Survival, OR = Odds Ratio, HR = Hazards Ratio, CI = Confidence Interval, ELN = European Leukemia Net 2017 prognostic risk classification, univar. = univariable model. Leukocytosis defined as WBC count >11 × 10^9^/L.

Our lab recently performed RNA-sequencing (RNAseq) of KG-1 cells with stable shRNA-mediated FOXM1 knockdown [6]. We have since used these RNAseq results to define a FOXM1 transcriptional signature comprising genes with |log2 fold change (FC)| > 10 when comparing expression in FOXM1-deficient cells to control scramble vector transduced cells. While 407 genes from this KG-1 cell line experiment met our fold change criterion, 362 of the genes had normalized RNA expression values in Beat AML patients [12]. We proceeded to examine these 362 genes within a cohort of *n* = 194 de novo AML patents with a wild-type FLT3 (FLT3^wt^) genomic profile and available Beat AML RNAseq data. The analysis was restricted to FLT3^wt^ AML as we have previously shown that deleterious FLT3-ITD mutations can activate FOXM1 directly through AKT signaling [6]. An unclustered heatmap (Fig. 1A) of RNA expression values (conditional quantile normalized and gene [row] standardized) shows the subset of differentially expressed genes between NPM1 mutant (*n* = 44) and NPM1 wild type (*n* = 150) patient samples – defined as NPM1^mut^/NPM1^wt^ |log2 FC| >1. The heatmap is ordered by NPM1 status fold change with the 93 genes downregulated in NPM1^mut^ compared to NPM1^wt^ listed above and the 32 genes upregulated in NPM1^mut^ AML. This heatmap shows an overall downregulation of FOXM1 activity in NPM1 mutant AML patient samples and likely represents the subset of FOXM1 target genes that contribute to the favorable outcome conferred by the NPM1 mutation (Fig. 1A). A FOXM1 transcriptional signature score was calculated for each Beat AML patient as the first principal component (i.e., eigengene) of conditional quantile (accounting for library size, GC content, and gene length) and specimen type normalized RNAseq read counts of the previously described 362 genes. These FOXM1 scores were significantly lower in NPM1^mut^ patients compared to NPM1^wt^ patients (Fig. 1B) demonstrating for the first time that NPM1 mutations are determinants of FOXM1 transcriptional activity. We then examined the prognostic relevance of FOXM1 transcriptional activity limiting the analysis to patients treated with intensive chemotherapy (*n* = 168). Overall survival (OS) following intensive chemotherapy was compared between 3 groups, NPM1^mut^ AML patients (*n* = 39) and NPM1^wt^ patients (*n* = 129) stratified into FOXM1-low and FOXM1-high groups based on median FOXM1 score. The Kaplan Meier survival curves demonstrate OS in FOXM1-low (≤ median) NPM1^wt^ patients was superior to the FOXM1-high (> median) NPM1^wt^ patients and approached the survival curve of the favorable subset of NPM1^mut^ AML patients (3-group log rank test *p* = 0.010) (Fig. 1C). The pairwise OS comparison between the two NPM1^wt^ groups (high vs. low FOXM1 transcriptional score) had HR = 1.55 (95% CI: 0.92–2.61) and Wald test *p* = 0.097 from a univariable Cox model (data not shown). A multivariable analysis was conducted controlling for well-validated patient and disease characteristics known to correlate with AML prognosis (e.g., age, ELN risk group). A high FOXM1 score was associated with inferior overall survival rate,OS (HR = 1.52, *p* = 0.139) and a two-fold increased risk of a disease-related death, disease-specific survival (HR = 2.02, *p* = 0.039) among the subgroup of patients with NPM1^wt^ treated with standard induction chemotherapy (Fig. 1D). Importantly, again in the multivariable setting, a high FOXM1 score was associated with over 60% reduction in the odds of achieving a composite complete remission after induction therapy among these NPM1^wt^ patients (OR = 0.37, *p* = 0.041) (Fig. 1D). These findings establish that low FOXM1 activity is an independent predictor of chemotherapy response and disease-specific survival for AML patients. The current data represent the first clinical validation that the FOXM1 transcriptional signature is linked to NPM1 mutational status and modulates treatment outcomes thereby making it an attractive target.

**Fig. 2:**
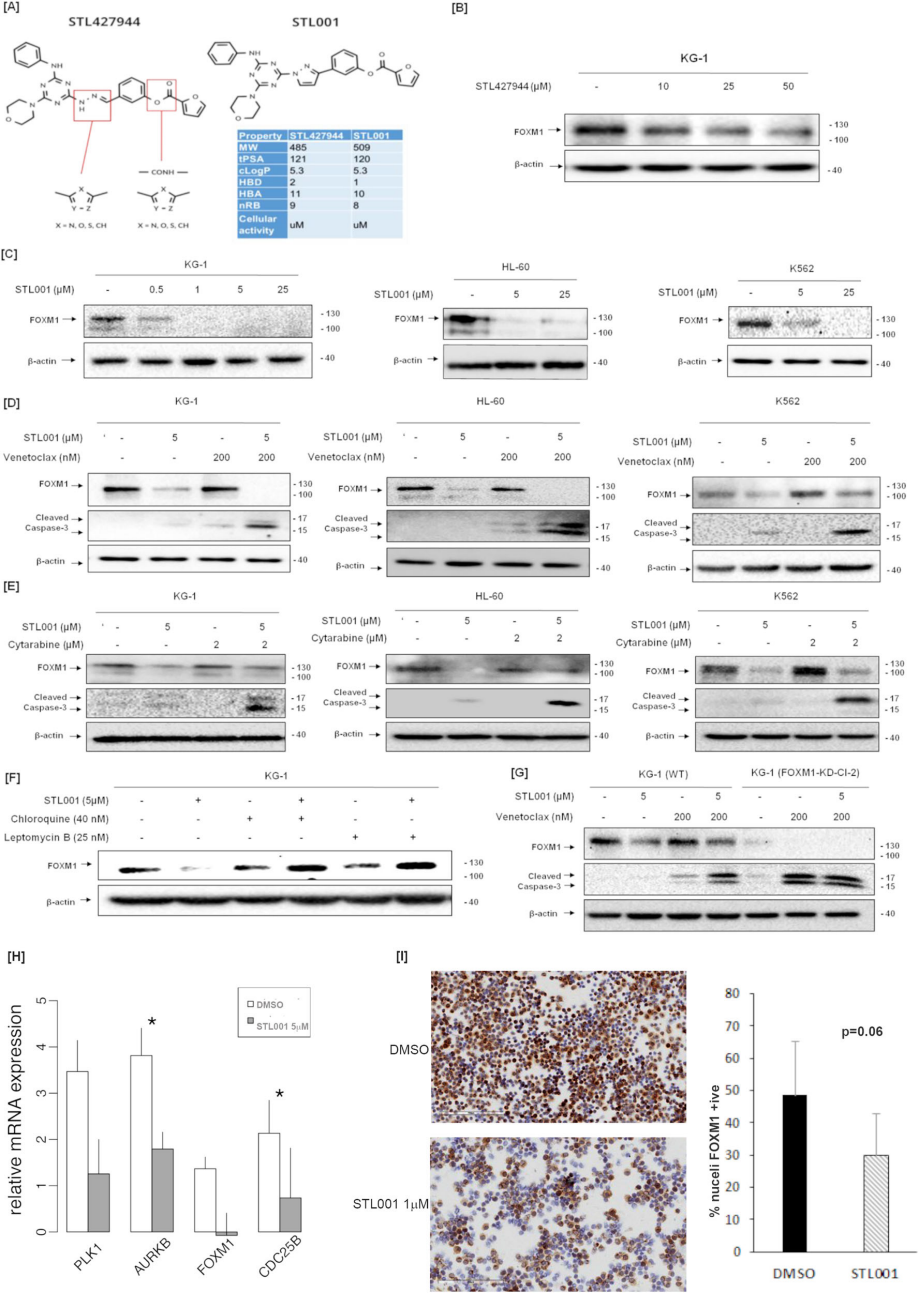
STL001 is a novel FOXM1 inhibitor that sensitizes leukemia cells to standard cytotoxic and bcl2 inhibitor therapies. **A** Structural formula of novel FOXM1 inhibitor STL001 and its precursor molecule STL427944. **B** AML cell line KG-1 treated with precursor compound STL427944. **C** AML cell lines KG-1, HL-60, and K562 were treated with increasing concentrations of STL001 for 24 h. Total protein samples obtained from treated cells were analyzed for FOXM1 protein levels using immunoblotting; β-actin was used as the internal loading control. **D** KG-1, HL-60, and K562 cells were treated with indicated concentrations of venetoclax and STL001 alone or in combination for 24 h. **E** KG-1, HL-60, and K562 cells were treated with indicated concentrations of cytarabine and STL001 alone or in combination for 24 h. In all cases, total protein samples were obtained from cells treatment and analyzed for FOXM1 and cleaved caspase-3 levels via immunoblotting; β-actin was used as internal loading control. **F** KG-1 cells were treated with indicated concentrations of Leptomycin B, Chloroquine, and STL001 for 24 h. Total protein samples obtained from treated cells were analyzed for FOXM1 protein levels via immunoblotting; β-actin was used as an internal loading control. **G** KG-1 cell lines with shRNA knockdown of FOXM1 were treated with venetoclax and STL001 and compared to parental cells (**H**) Real-time PCR-based expression profiling for canonical FOXM1 target genes was performed on primary AML mononuclear cells (*n* = 5) treated ex vivo with STL001 5 µM for 24 h. Mean and standard error of the delta Ct for DMSO or treatment group are plotted for each gene. *P*-value is estimated by two-tailed paired t-test. Asterisks denote *P* < 0.05. **I** Treatment of peripheral blood mononuclear cells with STL001 for 24 h followed by immunohistochemistry for FOXM1. Slides were scanned on an Aperio AT2 brightfield scanner and images were analyzed using HALO 2.0 software. Hematoxylin counterstain was used to segment nuclei within the ROIs and to establish an accurate cell count. Representative images are shown (5X) and % cells with nuclear FOXM1expression compared between DMSO and STL001 treated cells. *P*-value is based on one-tailed paired t-test.

We postulate that FOXM1 inhibitors will suppress FOXM1 function, thus mimicking the effect of the NPM1 mutation. Our group has previously described STL427944, a first-in-class small molecule inhibitor of FOXM1 [13] that was identified using a network-centric approach and confirmed using RNAseq data in cancer cell lines, as a selective FOXM1 inhibitor at very high concentrations. We applied several paths of structural activity relationship optimization and, out of 10 newly designed compounds, STL001 showed up to 50-fold increased potency as a FOXM1 inhibitor. The ring replacement is likely to have significantly improved the metabolic stability of the predecessor molecule, resulting in a more cellular active compound with better ‘drug-like’ properties. The initial modification of STL427944 (STL001) that increases its stability, thereby significantly improving potency, is shown in Fig. 2A. Upon treating a panel of AML cell lines – including KG-1, HL-60, and K562—with increasing concentrations of STL001 for 24 h, we observed a dose-dependent inhibition of FOXM1 protein expression at significantly lower concentrations (as low as 500 nM) compared to the precursor compound (STL427944) that showed modest FOXM1 inhibition at concentrations of 25 µM (Fig. 2B, C). STL001 treatment did not exert prominent cytotoxic action on its own, but when used in combination with either venetoclax (Fig. 2D) or cytarabine (Fig. 2E) in KG-1, HL-60 and K562 cells led to potent induction of apoptosis indicated by caspase-3 cleavage, providing a rationale for combination therapy with these drugs. The addition of Leptomycin B or Chloroquine rescued FOXM1 protein levels from suppression by STL001 (Fig. 2F), suggesting that STL001 induces cytoplasmic re-localization of FOXM1 and subsequent autophagy-dependent degradation. These results indicate that the first-generation modification drug, STL001, preserves the mode of action of the parent compound, STL427944, and potentially has the same high selectivity towards FOXM1. Moreover, this treatment sensitization effect of STL001 is absent in KG-1 cells with stable knockdown of FOXM1 (Fig. 2G), suggesting that the key effect of the compound on chemosensitization is through FOXM1 inhibition. We validated these findings in primary AML samples (*n* = 5) where ex vivo treatment with STL001 followed by real-time PCR analysis showed significantly suppressed expression of canonical FOXM1 transcriptional targets AURKB and CDC25B (Fig. 2H) in addition to downregulation of PLK1 and FOXM1 itself, due to a previously published autoregulation loop. In addition, primary AML samples (*n* = 4) were treated ex vivo with STL001 and slides were scanned on an Aperio AT2 brightfield scanner and images were analyzed using HALO 2.0 software. The % of nuclei staining positive for FOXM1 was calculated per cell and averaged per slide to analyze FOXM1 protein expression and localization. Results confirmed a decrease in nuclear FOXM1 protein levels after STL001 treatment (Fig. 2I) and approached statistical significance (43% vs. 29% of nuclei expressing FOXM1, paired t-test, one-tailed *p* = 0.06).

Overcoming resistance to chemotherapeutic drugs and BCL2 inhibitors represents a cornerstone issue in improving survival outcomes in AML. While there has been tremendous progress in understanding the oncogenic function of NPM1 mutations in AML [14, 15], little insight has been made into the fortuitous sensitization to chemotherapy and BCL2 inhibitors conferred by this mutation. The suppressed FOXM1 transcriptional activity in NPM1^mut^ AML patients in the current work brings to light a potential avenue to uncouple the oncogenic from the chemosensitizing effect of this highly prevalent mutation. Here, we show for the first time that FOXM1 transcriptional activity in AML patient samples can determine response to chemotherapy and independently predict disease-related death. Collectively our previous and current data suggest FOXM1 is an actionable target for AML patients with wild-type NPM1. Pharmacologic inhibition of FOXM1 may recapitulate the effects of the NPM1 mutation through the inactivation of FOXM1 and, thereby, confer more favorable treatment outcomes to NPM1^-^wild type AML patients with a current, dismal median survival of less than 1 year.
